# Evaluation and treatment of an unusual urachal mass: a case report

**DOI:** 10.1186/s40064-014-0782-9

**Published:** 2015-01-14

**Authors:** John L Cooper, Nikolai A Sopko, Trinity J Bivalacqua

**Affiliations:** James Buchanan Brady Urological Institute, The Johns Hopkins University School of Medicine, 600 North Wolfe Street/ Marburg 1, Baltimore, MD 21287 USA

**Keywords:** Urachal mass, Fistula, Diabetes, Cystectomy, Urachal abscess, Urachal adenocarcinoma

## Abstract

Abnormalities of the urachus, the vestigial remnant of the allantois, result when the embryonic lumen fails to completely obliterate during fetal development. In adults, urachal abnormalities are most commonly masses, with urachal adenocarcinoma representing the most frequent etiology. Due to the low incidence of urachal masses, guidelines for diagnostic workup and treatment are based off of a limited body of evidence comprised primarily of case reports and retrospective series. We present the case of a fifty-two-year-old woman with a urachal mass. Full radiologic workup consisting of computed tomography, cystoscopy and ultrasonography is included, and the risk factors, treatment and prognosis are discussed.

## Introduction

Urachal masses are exceedingly rare in the general population and can arise from a wide variety of causes. One retrospective epidemiological study of 33 adult patients with urachal masses found that 67% of cases were caused by carcinoma, most commonly adenocarcinoma, while the remaining 33% were due to benign etiologies including abscesses and cysts (Tian et al. 2008). Urachal adenocarcinoma comprises approximately 0.34% of all bladder neoplasms with only several hundred cases reported in the literature (Pinthus et al. 2006; Manunta et al. 2005). Unfortunately, the prognosis associated with urachal adenocarcinoma is poor, with 5-year disease-specific survival ranging from 40% to 61.3% underscoring the need for accurate diagnosis to properly treat and counsel the patient (Siefker-Radtke et al. 2003; Ashley et al. 2006). We report the evaluation and treatment of a urachal mass in a fifty-two-year-old woman and discuss the risk factors, treatment rationale and implications for her prognosis.

## Case presentation

**Figure 1:**
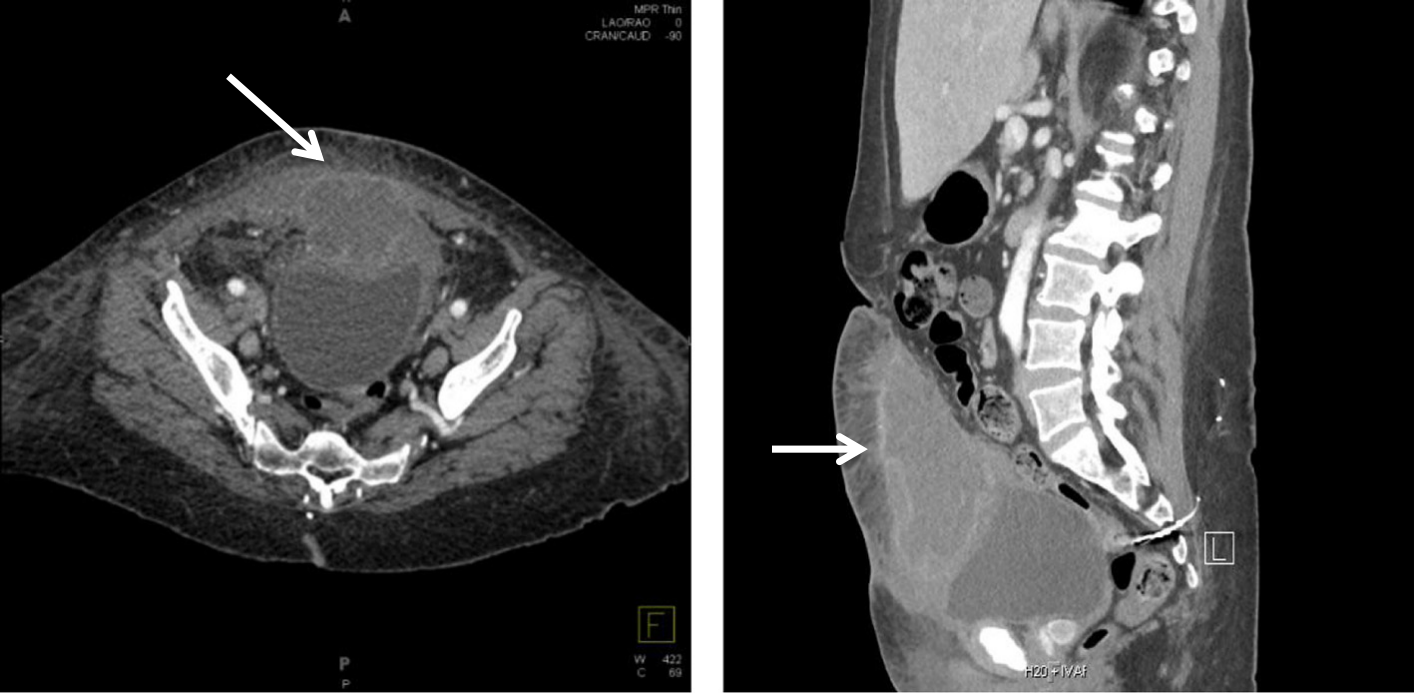
**CT scan of the abdomen and pelvis, axial (left) and sagittal (right) views.** The mass can be seen extending anteriorly and superiorly from the dome of the bladder (arrows).

A fifty-two-year-old woman with a history of poorly controlled Type II Diabetes Mellitus and depression presented to the Emergency Department with a 1-day history of fevers to 39.2°C and was found to be in diabetic ketoacidosis. During her stay in the ICU, she began to complain of pain below her umbilicus. She denied the presence of hematuria, umbilical drainage, or any other urinary symptoms. Her surgical history included a hysterectomy in the 1980’s and sacral nerve stimulator placement in the early 2000’s. On physical examination she was found to have a large rash in her perivaginal area and a small, palpable mass with induration and tenderness in the location of her periumbilical pain. A well-healed surgical scar from her prior hysterectomy was noted at the site of pain, but there was no erythema or drainage present. A CT scan of her abdomen and pelvis was obtained, which demonstrated a 5.3 × 8.8 × 12.6 cm rim-enhancing, loculated soft-tissue mass extending from the anterior dome of the bladder to the lower anterior abdominal wall and invading the rectus abdominis muscles, concerning for urachal carcinoma vs. urachal cyst abscess (Figure 1).

**Figure 2:**
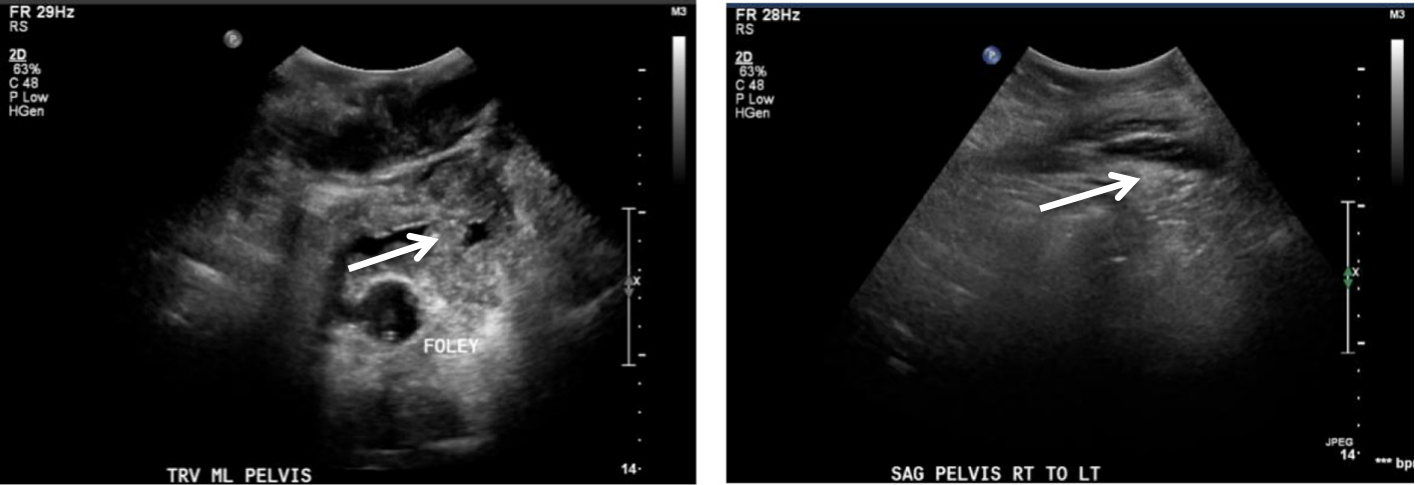
**Ultrasonography of the upper pelvis, transverse (left) and sagittal (right) views.** The heterogeneous mass (arrows) lies superficial to the bladder, which is decompressed with a Foley catheter.

Ultrasonography further characterized the mass as heterogeneous with both cystic and solid components, and revealed that there was not a drainable pocket (Figure 2). An ultrasound-guided fine needle aspiration was obtained for gross pathology, cytopathology and microbiologic analysis of the mass. These studies demonstrated marked acute inflammation, rare amounts of *Candida krusei* present*,* no evidence of bacteria, and no cytological or gross evidence of malignancy. Her blood and urine cultures since admission had been repeatedly negative for enteric bacteria or fungi, and her fever had dissipated.

At this time it was unclear whether the biopsy results from the mass demonstrated a fungal abscess, contamination from the skin or her pelvic rash, or a superinfected urachal adenocarcinoma. Infectious Diseases recommended empiric antibiotic coverage against gram-negative enteric flora with ciprofloxacin and metronidazole. She then underwent cystoscopy as an outpatient to evaluate for the presence of a vesico-urachal fistula. Significant purulent drainage was seen exuding from the dome of the bladder with surrounding areas of bullous edema Biopsy of the areas demonstrated chronic inflammation and polypoid cystitis, and the drainage of the mass was attempted.

**Figure 3:**
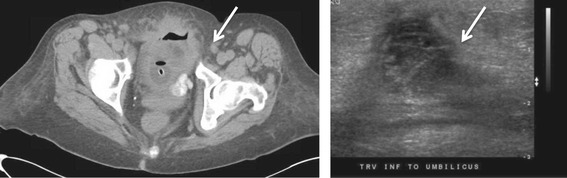
**CT scan (left) and Ultrasound (right) of the pelvis.** These studies demonstrate a smaller, heterogeneous mass (arrows) with a solid superior compartment and a walled-off, phlegmon-filled inferior compartment.

Repeat CT and pelvic ultrasound were performed to reassess the size and characteristics of the mass and further evaluate the location and presence of the fistula tract. These studies revealed that the mass was significantly smaller with maximal dimension of 3.7 cm, and the vesico-urachal fistula tract was visualized extending from the inferior portion of the mass to the bladder. More distinct regions were seen within the mass; the superior aspect had the heterogeneous appearance of a solid tumor with central liquefactive necrosis while the inferior region was consistent with a mature abscess (Figure 3).

Based on the results of these imaging studies, the etiology of the mass was thought to be either a superinfected urachal adenocarcinoma with bladder involvement and secondary abscess formation or a primary urachal abscess with fistula. She was placed on intravenous antibiotics and antifungals without clinical improvement. She subsequently underwent surgical removal of the mass, umbilicus and abdominal wall with partial cystectomy as a joint case between Urology and General Surgery. Tissues from the mass, bladder and abdominal wall sent for pathologic analysis revealed acute and chronic inflammation, a soft tissue fistula tract with fibrosis, focal abscess formation and no evidence of malignancy. At this time the final diagnosis of a fungal-infected urachal sinus was made, and she was given a peripherally inserted central catheter and placed on an outpatient regimen of Micafungin and Ertapenem for antifungal and antibacterial coverage. She was followed up post-operatively for two months in clinic by both Urology and General Surgery, and all drains and catheters were removed with no post-surgical complications.

## Discussion

Urachal masses have a very low prevalence in the population and are rarely seen in clinical practice. Thus, there are few guidelines for the evaluation, diagnosis and treatment of these cases. Although the majority of masses are malignant, benign causes are also seen including abscess as in the above case. It is important to understand how to discern between benign and malignant etiologies in order to promptly initiate proper treatment, reduce the performance of unnecessary surgeries in benign, non-infectious cases, and accurately inform patients of their prognosis. However, making a definitive pre-operative diagnosis of urachal masses is often difficult, due in part to the sparse amount of data describing the typical clinical and radiologic findings that could be used to distinguish between benign and malignant causes. In fact, one study demonstrated that no diagnostic test was accurate enough to preclude the surgical removal of a urachal mass of unknown etiology (Meeks et al. 2013).

Certain presenting symptoms have been shown to be more common in benign versus malignant urachal masses. While the most frequent symptom associated with urachal carcinoma is gross hematuria, benign etiologies, including abscesses and cysts, most commonly present with a palpable abdominal mass (Tian et al. 2008; Manunta et al. 2005; Ashley et al. 2006). There have been several similar cases reported in the literature of urachal abscesses that mimicked neoplasms on physical exam, CT and ultrasound, all of which were able to be diagnosed only following partial cystectomy and en bloc mass resection (Chen et al. 1992). A retrospective study described the most common features on CT scans of patients with proven cases of urachal adenocarcinoma to determine whether malignant cancers could be distinguished radiographically from benign or infectious masses. The majority of these tumors were characterized on CT as midline, supravesicular, mixed solid and cystic, calcified and frequently invading the bladder wall (Thali-Schwab et al. 2005).

These results help explain the difficulty and complexity of the diagnosis in the above case, as the patient’s mass was midline, supravesicular and had a mixed solid and cystic appearance. However, this patient has several additional risk factors for having a fungal abscess rather than adenocarcinoma, most notably a history of uncontrolled diabetes, a pelvic fungal rash, and a midline periumbilical surgical scar that could have served as a portal of entry for her infection. Follow-up for urachal masses is dependent on the etiology. Malignant causes require extensive follow-up, whereas non-malignant causes removed surgically are generally considered resolved once removed and generally do not require additional follow-up other than that expected for post-operative care.

Ultimately, the final diagnosis of a benign versus malignant urachal mass has implications on treatment and overall outcomes. Urachal adenocarcinoma carries a particularly grave prognosis in most circumstances (Pinthus et al. 2006; Siefker-Radtke et al. 2003; Ashley et al. 2006). It is important to properly inform patients with cancer of the reality of their disease, while alleviating the concerns of those patients with a more benign etiology. Standard therapy for either malignant or benign urachal masses based on both prospective and retrospective evidence includes en bloc resection with or without partial cystectomy (Herr et al. 2007). The need for adjuvant therapies and long-term follow-up varies depending on the etiology (Meeks et al. 2013; Herr et al. 2007). Patients with adenocarcinoma require additional evaluation for disease monitoring, whereas this patient required an intravenous antifungal regimen and post-operative follow-up.
